# The Role of Preoperative Bilateral Breast Magnetic Resonance Imaging in Patient Selection for Partial Breast Irradiation in Ductal Carcinoma *In Situ*

**DOI:** 10.1155/2012/206342

**Published:** 2012-05-13

**Authors:** Kristin V. Kowalchik, Laura A. Vallow, Michelle McDonough, Colleen S. Thomas, Michael G. Heckman, Jennifer L. Peterson, Cameron D. Adkisson, Christopher Serago, Steven J. Buskirk, Sarah A. McLaughlin

**Affiliations:** ^1^Department of Radiation Oncology, Mayo Clinic, 4500 San Pablo Road, Jacksonville, FL 32224, USA; ^2^Department of Radiology, Mayo Clinic, 4500 San Pablo Road, Jacksonville, FL 32224, USA; ^3^Biostatistics Unit, Mayo Clinic, 4500 San Pablo Road, Jacksonville, FL 32224, USA; ^4^General Surgery, Mayo Clinic, 4500 San Pablo Road, Jacksonville, FL 32224, USA

## Abstract

*Purpose*. Women with ductal carcinoma *in situ* (DCIS) are often candidates for breast-conserving therapy, and one option for radiation treatment is partial breast irradiation (PBI). This study evaluates the use of preoperative breast magnetic resonance imaging (MRI) for PBI selection in DCIS patients. *Methods*. Between 2002 and 2009, 136 women with newly diagnosed DCIS underwent a preoperative bilateral breast MRI at Mayo Clinic in Florida. One hundred seventeen women were deemed eligible for PBI by the NSABP B-39 (National Surgical Adjuvant Breast and Bowel Project, Protocol B-39) inclusion criteria using physical examination, mammogram, and/or ultrasound. MRIs were reviewed for their impact on patient eligibility, and findings were pathologically confirmed. *Results*. Of the 117 patients, 23 (20%) were found ineligible because of pathologically proven MRI findings. MRI detected additional ipsilateral breast cancer in 21 (18%) patients. Of these women, 15 (13%) had more extensive disease than originally noted before MRI, and 6 (5%) had multicentric disease in the ipsilateral breast. In addition, contralateral breast cancer was detected in 4 (4%). *Conclusions*. Preoperative breast MRI altered the PBI recommendations for 20% of women. Bilateral breast MRI should be an integral part of the preoperative evaluation of all patients with DCIS being considered for PBI.

## 1. Introduction

Ductal carcinoma *in situ* (DCIS) is a noninvasive breast cancer and represents a complex pathologic condition in which malignant epithelial cells arise and proliferate within the ducts of the breast but do not invade the basement membrane. According to the Surveillance Epidemiology and End Results program (SEER), DCIS represents 14% of all new breast cancer diagnoses in the United States [1].

Radiation therapy has historically been delivered to the whole-breast after breast-conserving surgery. Adjuvant radiation has been shown to improve local tumor control in multiple prospective, randomized clinical trials [2–4]. Partial breast irradiation (PBI) has been developed as a way to deliver radiation directly to the tumor cavity of the breast after breast-conserving surgery in lieu of whole-breast radiation therapy. PBI can be delivered by multiple techniques, including interstitial and intracavitary brachytherapy, intraoperative radiotherapy, 3-dimensional (3D) conformal or intensity-modulated radiation therapy, or proton therapy. As less breast tissue is being irradiated, the potential benefits include decreased acute toxicity to the breast and potential decreased risk of late toxicity due to reduced radiation dose to the surrounding tissue [5]. This reduced risk includes the potential for decreased heart and lung toxicity [6]. An additional benefit to patients is the decreased total treatment time. One commonly used course of PBI is 34 gray administered twice daily over 5 days, for a total of 10 fractions. Multiple fractionation schemes have been used, including single-fraction treatments. For a select group of patients, equivalent results have been reported for PBI versus whole breast external beam radiotherapy [5, 7, 8].

 The National Surgical Adjuvant Breast and Bowel Project, Protocol B-39 (NSABP B-39) is a prospective randomized trial in which eligible women with early-stage breast cancer are randomized to whole-breast radiation therapy versus PBI. Specific eligibility criteria include tumor size ≤3 cm, ≤3 positive lymph nodes, negative surgical margins, lack of multicentric disease, and no contralateral breast cancer.

Bilateral breast magnetic resonance imaging (MRI) is being used preoperatively with increasing frequency for women with a new diagnosis of breast cancer. MRI has been found to enhance findings in women when performed after an initial clinical evaluation. Specifically in DCIS, MRI has been prospectively shown to have a sensitivity of 92%, and up to 98% for high-grade DCIS [9]. Multiple meta-analyses, which include DCIS patients, have shown that MRI of the ipsilateral breast detects additional disease in 16–20% of women with newly diagnosed breast cancer [10, 11]. After an initially negative evaluation, contralateral breast cancer is detected by MRI in 3% to 6% of patients [11, 12]. These results have been shown to alter surgical recommendations [10–12].

This study evaluates the role of bilateral breast MRI in determining eligibility for PBI based on the NSABP-B39 criteria in women with newly diagnosed DCIS.

## 2. Methods

This is a retrospective review of women diagnosed with DCIS at Mayo Clinic in Florida between 2002 to 2009. All women with a new diagnosis of DCIS who underwent a preoperative bilateral breast MRI, regardless of their ultimate treatment, were included in this study. Data collected on these women included patient demographics, tumor characteristics, MRI findings, and pathologic information. At Mayo Clinic in Florida a preoperative bilateral breast MRI is recommended as part of the evaluation of all women with a new diagnosis of DCIS.

Women with DCIS were initially evaluated after the standard clinical evaluation, which consisted of physical examination, mammogram and/or ultrasound, and pathologic examination of a tissue biopsy. On the basis of the initial clinical evaluation, each patient was determined to be eligible or ineligible for PBI according to the NSABP B-39 criteria. These criteria included tumor size ≤3 cm (including multifocal tumors to a maximum extent of 3 cm); negative final surgical margins; lack of multicentric disease; no contralateral breast cancer. Multicentric disease was defined as additional disease >4 cm from the original tumor volume or disease within a different breast quadrant. Negative surgical margins are defined as histologically free of invasive and noninvasive tumor. Each patient was reviewed again after the bilateral breast MRI, and the eligibility for PBI was reassessed. All changes in PBI recommendations made on the basis of MRI findings were confirmed by final pathology. Tumor specimens were histologically evaluated, and final DCIS tumor size was determined by the maximum histologic size. Patients were excluded from analysis if any of these criteria were not met.

 Breast MRI examinations were performed with the patient in the prone position in a 1.5-tesla system (Avanto, Espree, Sonata, or Symphony; Siemens Medical Solutions USA Inc., Malvern, PA), using a dedicated surface breast coil. The imaging sequence included an axial pre-contrast 3D fast low-angle shot (FLASH) sequence, T1-weighted, 3D, gradient-echo scans of both breasts, followed by a sagittal T2-weighted turbo spin echo sequence of each breast. Slice thickness was 3 mm with a 0.6 mm gap for all images. The next series of images obtained included sagittal 3D flash images of the breast with known cancer, then imaging of the contralateral breast using the same technique, both before and after a bolus of intravenous gadodiamide (Omniscan; GE Healthcare, Waukesha, WI). If the patient's glomerular filtration rate was higher than 60 mL/min, 20 mL of gadobenate dimeglumine (MultiHance; Bracco Diagnostics Inc,, Princeton, NJ) was used, and if the glomerular filtration rate was 30 to 60 mL/min, 15 mL of gadobenate dimeglumine was administered. Patients with a glomerular filtration rate <30 mL/min were not given a contrast agent unless it was deemed absolutely necessary. Finally, bilateral axial postcontrast 3D FLASH images were obtained using the above-described parameters. Postprocessing included subtraction of the pre- and postcontrast images and motion correction, if necessary. MRIs were interpreted by board-certified, subspecialist breast radiologists.

 Patients who were deemed ineligible for PBI on the basis of their bilateral breast MRI were categorized by the reasons for their ineligibility. Specifically, women were ineligible if the size of the tumor was described on MRI (and pathologically proven) to be >3 cm. Also ineligible were patients with multifocal disease if the total tumor extent was >3 cm. These patients were classified as having more extensive disease than determined by initial clinical evaluation. Patients were also deemed ineligible if they were diagnosed with multicentric breast cancer, which was defined as additional disease >4 cm from the original tumor volume or disease within a different quadrant. If any cancer diagnosis was made in the contralateral breast, the patient also was deemed ineligible. Results were confirmed postsurgically with the final pathologic findings.

## 3. Statistics

Characteristics of patients, final pathology results, and MRI findings were summarized by sample median, 25th percentile and 75th percentile for numerical variables, and by number and percentage for categorical variables. For evaluation of the primary aim, the proportion of DCIS patients whose eligibility for PBI was altered by bilateral breast MRI findings was estimated along with an exact binomial 95% confidence interval (CI). All analyses were performed using SAS (version 9.2; SAS Institute Inc., Cary, NC).

## 4. Results

Between January 2002 and June 2009, 136 women with newly diagnosed DCIS underwent a preoperative bilateral breast MRI. Of these patients, 117 women (86%) were deemed eligible for PBI based on the NSABP-B39 inclusion criteria after their initial clinical evaluation (i.e., a physical examination, mammogram and/or ultrasound, and pathologic findings). Characteristics of the 117 women (median age, 63; range 36–90) who were initially eligible for PBI are summarized in Table 1.

The pathologic results of the women who were recommended to undergo a biopsy following MRI were evaluated. In this cohort, 39% of women were recommended to have a biopsy either of the ipsilateral, contralateral, or bilateral breasts. Of these patients, 37% were found to have additional cancer.

**Figure 1:**
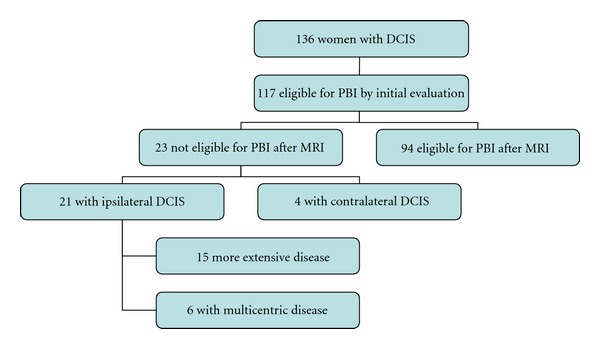


MRI findings led to pathologically proven additional disease that altered the recommendations for PBI in 23 (20%; 95% CI: 13%–28%) of the 117 patients initially eligible for PBI. MRI detected additional ipsilateral breast cancer in 21 (18%) patients. Of these women, 6 (5%) were diagnosed with multicentric breast cancer. A total of 15 (13%) women became ineligible for PBI due to more extensive disease as determined by MRI and confirmed by pathologic results. Contralateral breast cancer was detected in 4 (4%) women on the basis of MRI results (Figure 1). Two women were found to have both additional ipsilateral disease and contralateral breast cancer.

Table 2 summarizes the final pathologic findings of the 117 women initially deemed appropriate for PBI, and of the 23 women ultimately ineligible for PBI. Within this group, 8 (7%) patients were ultimately diagnosed with invasive breast cancer.

A total of 15 patients had more extensive disease and the median tumor size in this group was 4.0 cm. Of the 6 patients with multicentric disease, all additional tumors were smaller than 1 cm, and none of the patients had more than 2 tumors. The 4 patients with contralateral tumors detected by MRI were all smaller than 2 cm.

## 5. Discussion

This study evaluates the use of bilateral breast MRI as a diagnostic tool for women with newly diagnosed DCIS being considered for PBI following breast conserving surgery. Of 117 patients deemed eligible for PBI after their initial clinical evaluation, 20% became ineligible after MRI. Reasons for ineligibility included additional findings of more extensive disease, multicentric breast cancer, or contralateral breast cancer.

Previous studies have focused on invasive cancers exclusively, or in combination with non-invasive cancers. This is the first published study evaluating the role of MRI in determining patient eligibility for PBI exclusively in patients with DCIS. Previous studies, including our unpublished data, show a change of 2–11% in PBI recommendations for all breast cancer patients with a diagnosis of multicentric disease after breast MRI [13–18]. Multifocality has also been evaluated with a range of 4–7% change in PBI recommendations. Contralateral breast cancer diagnoses after MRI have produced a 2–5% change in PBI recommendations [13–18].

This study found 5% of patients had a change in PBI recommendations after MRI because of additional findings of multicentric disease. This correlates with the published data, as does the 4% change in PBI recommendations after MRI for contralateral disease. This study did not examine only multifocal disease, but it did evaluate multifocal disease that led to ineligibility, as well as disease larger on MRI and confirmed by pathology, which was defined as more extensive disease. Therefore, the percentage of patients with altered treatment recommendations because of more extensive disease (13%) was greater than that in published reports from previous studies that evaluated multifocal disease alone.

These results for DCIS correlate well with those reported in the medical literature on additional diagnoses made by MRI. About one-fifth (18% (21/117) of the patients had altered eligibility either because of multicentric disease, multifocal disease, or larger disease within the ipsilateral breast, which correlates well with the 16–20% additional ipsilateral breast cancer diagnoses reported in the MRI literature [10, 11]. The 4% of patients in whom a contralateral breast cancer was diagnosed also correlates well [11, 12]. It is important to note that of the 20% of patients ineligible for PBI, not all of these women would have been excluded from consideration of breast-conserving surgery.

All the patients included in this study underwent an MRI on a 1.5-T machine. Plana et al. found a statistically significant higher positive predictive value for breast MRI when using ≥1.5 T MRI [11]. MRI has also been found to have a high sensitivity for DCIS as reported by Kuhl et al. with 1.5T machines [9]. Some previous studies used 1-T MRI, which may have led to decreased detection of additional disease.

This study is limited as a retrospective review. It is, however, the first study conducted to date that evaluates the role of MRI in determining appropriate candidacy for PBI for women with DCIS. It is also a more thorough evaluation about the utility of MRI for not only evaluating multicentric and contralateral breast cancers but also for evaluating more extensive disease, including tumors >3 cm and multifocal disease that extends >3 cm within the breast. By including more extensive disease, an additional 13% of women were identified as being ineligible for PBI after breast MRI than by evaluation of multicentricity and contralateral disease alone.

## 6. Conclusions

These results show that 20% of women with DCIS became ineligible for PBI after a bilateral breast MRI. Recognition of PBI ineligibility prior to surgery can improve clinical planning, including the avoidance of unnecessary procedures associated with brachytherapy and intraoperative radiation therapy. Bilateral breast MRI should be an integral part of the preoperative evaluation of all patients with DCIS who are being considered for PBI.

## Figures and Tables

**Table 1:** Characteristics of 117 women with DCIS eligible for partial breast irradiation on the basis of an initial clinical evaluation, and of the 23 women ultimately ineligible by MRI.

Variable	Overall (*N* = 117)	Ineligible based on MRI findings (*N* = 23)
	No. (%)^a^	
Age at diagnosis, y		
31–40	3 (3%)	1 (4%)
41–50	20 (17%)	4 (17%)
51–60	31 (26%)	4 (17%)
61–70	25 (21%)	4 (17%)
71–80	30 (26%)	7 (30%)
81–91	8 (7%)	3 (13%)
Race		
African American/Black	4 (3%)	1 (4%)
Asian	1 (1%)	0 (0%)
Caucasian	105 (90%)	21 (91%)
Hispanic	4 (3%)	1 (4%)
Other	3 (3%)	0 (0%)
Menopausal status		
Post	89 (76%)	16 (70%)
Pre	28 (24%)	7 (30%)
No. of first degree relatives with history of breast cancer		
0	85 (73%)	14 (61%)
1	23 (20%)	6 (26%)
2-3	8 (7%)	2 (9%)
Unknown/not available	1 (1%)	1 (4%)
No. of relatives with history of breast cancer		
0	71 (61%)	13 (57%)
1	27 (23%)	5 (22%)
2–4	18 (15%)	4 (17%)
Unknown/Not available	1 (1%)	1 (4%)
Dense breasts		
No	32 (27%)	8 (35%)
Yes	79 (68%)	14 (61%)
Not reported/indeterminate	6 (5%)	1 (4%)
Detection method		
Mammogram	105 (90%)	19 (83%)
Palpation	10 (9%)	3 (13%)
Other	2 (2%)	1 (4%)
Lumpectomy	68 (58%)	4 (17%)
No. of days from diagnosis to MRI	14 (0, 9, 23, 95)	12 (4, 8, 22, 34)
No. of days from diagnosis to surgery	38 (6, 27, 56, 813)	36 (21, 27, 57, 813)

^a^Sample median (minimum, 1st quartile, 3rd quartile, maximum) is given for numerical variables, whereas *n* (%) is given for categorical variables.

**Table 2:** Final pathological results for 117 women eligible for partial breast irradiation on the basis of the initial clinical evaluation, and of the 23 women ultimately ineligible by MRI.

Variable	Overall (*N* = 117)	Ineligible based on MRI findings (*N* = 23)
	No. (%)^a^	
Tumor size (cm)		
Not available	8 (7%)	0 (0%)
0.1-1.0	61 (52%)	6 (26%)
1.1–2.0	28 (24%)	3 (13%)
2.1–3.0	9 (8%)	3 (13%)
>3.0	11 (9%)	11 (48%)
Number of tumors (>1)	14 (12%)	8 (35%)
EIC (positive)	6 (5%)	4 (17%)
T stage		
Tis	109 (93%)	18 (78%)
T1a	6 (5%)	3 (13%)
T1b	1 (1%)	1 (4%)
T2	1 (1%)	1 (4%)
N stage		
NX	44 (38%)	5 (22%)
N0	70 (60%)	16 (70%)
N1	3 (3%)	2 (9%)
Lymphovascular space invasion		
No	113 (97%)	23 (100%)
Yes	1 (1%)	0 (0%)
Not reported/indeterminate	3 (3%)	0 (0%)
Lobular features	1 (1%)	1 (4%)
Grade		
Low	26 (22%)	10 (43%)
Intermediate	25 (21%)	2 (9%)
High	66 (56%)	11 (48%)
ER		
Negative	25 (21%)	8 (35%)
Positive	89 (76%)	14 (61%)
Not tested/not available	3 (3%)	1 (4%)
PR		
Negative	36 (31%)	10 (43%)
Positive	77 (66%)	12 (52%)
Not tested/not available	4 (3%)	1 (4%)

EIC: extensive intraductal component. ER: estrogen receptor. PR: progestin receptor.

^a^Values are numbers (percentage).
